# Food Safety Analysis of Milk and Beef in Southwestern Uganda

**DOI:** 10.1155/2018/1627180

**Published:** 2018-08-13

**Authors:** Keneth Iceland Kasozi, Phyllis Candy Natabo, Sarah Namubiru, Dickson Stuart Tayebwa, Andrew Tamale, Pwaveno H. Bamaiyi

**Affiliations:** ^1^Department of Physiology, Faculty of Biomedical Sciences, Kampala International University Western Campus, Box 71, Bushenyi, Uganda; ^2^Department of Public Health, School of Allied Health and Medicine, Kampala International University Western Campus, Box 71, Bushenyi, Uganda; ^3^College of Veterinary Medicine Animal Resources and Biosecurity, Makerere University, Box 7062, Kampala, Uganda; ^4^National Research Center for Protozoan Diseases, Obihiro University of Agriculture and Veterinary Medicine, Nishi 2 Sen-11, Inada-cho, Obihiro, Hokkaido 080-8555, Japan; ^5^Postgraduate School and Research Directorate, Kampala International University Western Campus, Box 71, Bushenyi, Uganda

## Abstract

**Background:**

Inorganic pollutants in milk and beef are of major public health concern; however, information in Africa is still limited due to low food safety monitoring practices. In this study, we established levels of lead (Pb), zinc (Zn), cadmium (Cd), copper (Cu), and iron (Fe) in milk and beef and obtained the estimated daily intake (EDI) and incremental lifetime cancer risk (ILCR) as measures of risk to the Ugandan population.

**Materials and Methods:**

This was a cross-sectional study in which a total of 40 samples of milk and beef were collected from Bushenyi district in southwestern Uganda. Samples were analyzed by atomic absorbance spectrophotometer, and the EDI and ILCR were computed using the US EPA reference values.

**Results and Discussion:**

Heavy metal concentrations were highest in the order of Zn > Fe > Pb > Cu in milk samples, while in beef samples, concentrations were highest in the order of Zn > Pb > Fe > Cu and no Cd was detected. Furthermore, beef had significantly higher (*P* < 0.05) Pb and Fe concentrations than milk. The EDI was highest in children, and this was followed by very high ILCR levels, showing that milk and beef are not safe for children in Uganda. Bearing in mind that a high HI was shown, beef and milk from these regions are not recommended for consumption especially by children although more studies remain to be conducted.

**Conclusion:**

Heavy metals in milk and beef of Uganda may predispose the indigenous community to cancer and other health-related illnesses, showing a need for improved food safety screening to promote food safety.

## 1. Introduction

Animal food product contamination with inorganic pollutants has increased due to intensified human activities and industrialization [1]. These inorganic pollutants are a threat to animal life as they affect key physiochemical processes in animal tissues due to their toxic effects [2, 3]. This has led to a strain on the livelihood and health of people, especially in the developing countries [4, 5]. In Africa, contamination of major water bodies with inorganic compounds such as lead (Pb), zinc (Zn), cadmium (Cd), iron (Fe), and copper (Cu) [6] has been documented [7] and is thought to be associated with the indiscriminate usage of pesticides in agricultural farming systems [8, 9]. Unfortunately, there is paucity of data due to lack of routine monitoring and reporting for the levels of inorganic pollutants in animal food products from developing countries [7, 10].

In Uganda, recent evidence has shown that roasted beef sold in Central Uganda has high levels of inorganic pollutants [11, 12]. This was a worrying discovery since majority of the beef supplied to Central Uganda comes from the cattle corridor districts, of which Bushenyi district is a part [13–15]. The consumption of contaminated milk and beef foods would subsequently lead to a buildup of heavy metals, leading to the development of health complications. On the other hand, some inorganic compounds such as Cu, Zn, and Fe are required for physiological functions in the body and must be present in microamounts [16]. In particular, zinc is important in key biodegradation mechanisms, especially in detoxification of the body through its antioxidative properties [17, 18]. Copper is also important in several enzymatic processes, and improved copper level regulation in the body has been associated with improved brain function [19]. Iron is a key component of red blood cells and also plays a crucial role in gene expression [20]. On the contrary, Pb and Cd are teratogenic [21, 22], while micronutrients once consumed in amounts beyond the international recommended levels may also lead to severe health effects.

In Uganda, Pb, Zn, and Cd continue to be key components of major agricultural products [23], and they have established cancer slope factors (CSF) [24]. This shows that the environment is routinely contaminated with inorganic compounds as a result of human activities probably as a result of poor regulatory policy. Pathological effects are bound to be observed in the body once human consumption of the heavy metals is beyond the international reference levels. However, while attempts to induce cancer by Zn, Cu, and Fe have been unsuccessful [25], Pb and Cd have established carcinogenic properties [26]. This is important since cancer risk estimation relies entirely on the cancer slope factor (CSF), which is often conducted in experimental animals and correlated to humans.

Food safety studies on milk and beef in Uganda had previously placed a lot of emphasis on microbial load [27, 28], leading to a scarcity of information on the inorganic pollutant load. This is important since human exposure to milk from cows that graze on heavy metal-polluted pastures has been associated with long-term health hazards such as cancer, organ damage, failure, and inflammation [29]. This makes research to determine the level of inorganic pollutants in animal products important, especially at the time when industrialization and chemical use have increased significantly. In addition, it showed that daily exposure of low amounts of inorganic compounds increases the estimated daily intake (EDI) [30], and since EDI is weight dependent, children have been shown to be more at risk than adults [31]. The EDI for lead in the Ugandan population eating roasted meat has already been shown to be above international recommended levels [11]. Not only can inorganic pollutants accumulate in the body to cause cancer but they can also disrupt normal body function once they build up in the tissues to toxic levels [32]. The target (which is the metal) hazard quotient (THQ) demonstrated the possibility of disastrous health effects developing in this regard. The objective of the study was to determine concentrations of major inorganic compounds in milk and beef and establish their safety for the Ugandan population. This was important since a majority of homesteads in Uganda rely on milk and beef of cattle origin as a reliable source of protein [13].

## 2. Materials and Methods

### 2.1. Study Area

This was a cross-sectional study in which milk and beef samples were collected from Bushenyi district. Bushenyi district lies in the cattle corridor of Uganda, and historically, it is associated with high milk and beef contributions to the Ugandan economy [13, 14]. Names of the study areas were entered in MS Excel and assigned a random number. These were then autoarranged and the first 5/9 subcounties of the district were included in the study to generate the sample areas.

### 2.2. Sample Collection

A total of 20 samples each of milk and beef were collected from 5 randomly chosen subcounties in Bushenyi district. In brief, samples were collected using sterile bottles for both milk (approximately 10 ml) and beef (approximately 200 g), transported under ice, and stored in a refrigerator at −20°C at the Department of Physiology, Faculty of Biomedical Sciences of Kampala International University Western Campus. These were subsequently transported frozen under ice to the Industrial Chemistry Laboratory in the School of Natural Sciences under Makerere University for Pb, Zn, Cd, Cu, and Fe analysis.

### 2.3. Sample Preparation

Sample preparation and analysis were conducted using standard methods [33]. In brief, the milk and beef samples were weighed in separate beakers. Wet digestion of the samples was subsequently done using 30 ml of nitric acid at 150°C for 45 minutes. The solution was left to evaporate up to 10 ml, and 2 ml of hydrogen peroxide was added followed by deionized water up to 30 ml. The solution was then transferred to a plastic bottle ready for analysis. The sample solution was analyzed with an atomic absorption spectrophotometer (AAS) (Perkin Elmer 2380).

### 2.4. Preparation of Standards

Working standards of 0.2 ppm, 0.5 ppm, 1 ppm, 2 ppm, and 5 ppm were prepared from stock solution of 1000 ppm (Pb, Cd, Cu, and Zn). The stock solutions for Pb were acquired from E. Merck, D-6100 Darmstadt, FR Germany. Analysis was done using AAS, and the respective absorbance for each metal was read and a standard calibration curve was generated for each pollutant. From the standard curves, equations were generated and used to determine the concentrations for the samples as shown below:

  Equation for Pb: absorbance (*y*) = 0.0092 × concentration (x), *R*^2^ = 0.9784
  Equation for Cd: absorbance = 0.0356 × concentration, *R*^2^ = 0.9494
  Equation for Cu: absorbance = 0.1109 × concentration, *R*^2^ = 0.9987
  Equation for Zn: absorbance = 0.4951 × concentration, *R*^2^ = 0.9987
  Equation for Fe: absorbance = 0.171 × concentration, *R*^2^ = 0.9318.

### 2.5. Determination of the Concentration of Heavy Metals

The homogenized samples were subjected to heavy metal analysis against Pb, Zn, Cd, Cu, and Fe. The absorbance for each sample was taken and the concentration determined in ppm using the equation generated from the standard curves.

### 2.6. Determination of the Estimated Daily Intake for the Ugandan Population

This was measured by using guidelines from the US EPA [24]. Estimation of daily intake (EDI) was measured according to methods using a weight of 60.7 kg for adults and 20.5 kg for children in line with global estimates for the Ugandan population [31].

EDI = (*C* × IR)/BW, where *C* = concentration of the metal (mg/kg), IR = ingestion rate for beef, and BW=beef weight. The ingestion rate of 120 g/day among adults and 56.7 g/day among children in Uganda was used [11]. A baseline consumption rate of milk of 63 ml/day for Uganda was used [34] and these were compared to the tolerable allowance levels [11].

### 2.7. Determination of the Cancer Risk for the Ugandan Population

The incremental lifetime cancer risk was used to measure the cancer risk in the Ugandan population. The chronic daily intake was first calculated using the following equation:

(1) $$\begin{array}{c} \text{CDI}=\frac{\left(\text{EDI}\times \text{EFr}\times \text{EDtot}\right)}{\text{AT}}, \end{array}$$

where EDI is the estimated daily intake of a metal via consumption of specific route, EFr is exposure frequency (365 days/year), EDtot is the exposure duration of 58.65 years (lifetime average for Ugandans), and AT is the period of exposure for noncarcinogenic effects (EFr x EDtot) and 70-year lifetime for carcinogenic effects (i.e., 70 years × 365 days/year) in line with international health projections for Uganda [31]. A 70-year period was used to reduce on the possibility of uncertainty since an exposure of 50% less would make the results less reliable [11].

(2) $$\begin{array}{c} \text{ILCR}=\text{CDI}\times \text{CSF,} \end{array}$$

where CDI is the chronic daily intake of a particular metal representing the lifetime average daily dose of exposure to a chemical. CSF of 0.0085, 0.0001, and 6.3 for Pb, Zn, and Cd was used, respectively [24].

### 2.8. Determination of Noncancer Risks for Ugandan Population

This was done using the following equation:

(3) $$\begin{array}{c} \text{THQ}=\frac{\text{CDI}}{\text{RfD}}, \end{array}$$

where THQ = target hazard quotient, CDI = exposure dose obtained, and RfD = oral reference dose of the contaminant, which is an estimation of the maximum permissible risk on human population through daily exposure. The reference dose (RfD) for each hazard obtained from the US EPA [35] was 0.004 ppm, 0.3 ppm, 0.001 ppm, 0.04 ppm, and 0.7 for Pb, Zn, Cd, Cu, and Fe, respectively.

### 2.9. Data Analysis

Data were entered in duplicates and transferred into Microsoft Excel version 2013 for analysis. Information was presented descriptively as mean ± SEM, and a two-sample *t*-test was conducted for comparisons on concentrations among adults and children for each metal with *P* < 0.05 taken as significant. EDI levels were determined for adults and children and compared to the tolerable allowable intake levels (TAL). ILCR for allowable and nonallowable levels were indicated by superscripts “*a*” and “*b*,” respectively, after comparing them to the US EPA reference values in the normal range of 10^−6^ to 10^−4^ [11, 36]. Finally, HI > 1 was taken as indicative of a threat [37].

## 3. Results

### 3.1. Levels of Heavy Metals in Milk and Beef of Bushenyi District

The heavy metal concentrations were generally in the order of Zn > Fe > Pb > Cu in milk samples, while in beef samples, concentrations were highest in the order of Zn > Pb > Fe > Cu, and no Cd was detected in both milk and beef samples. Mean concentrations of Zn and Cu in both the milk and beef samples were not significantly different (*P* > 0.05). In addition, significant differences (*P* < 0.05) were found to exist between Pb and Fe concentrations. In particular, Pb concentrations in beef were 18.90 ± 2.40 ppm, while in milk, it was 10.48 ± 1.82 ppm. Also, Fe levels were higher in beef than milk, that is, 17.04 ± 1.15 and 11.96 ± 1.00 ppm as shown in Table 1.

### 3.2. Estimated Daily Intake of Meat and Milk in Ugandan Population

The estimated daily intake (EDI) was generally all below the tolerable allowable levels except for lead in both adult and children. Furthermore, the EDI of zinc was highest in milk, especially in children, while that of copper in adults was the lowest. Furthermore, significant differences (*P* < 0.05) in the EDIs for milk were found to exist between adults and children as shown in Table 2.

### 3.3. Estimation of the Cancer and Noncancer Risk in Ugandan Population

The study showed that the incremental lifetime cancer risk (ILCR) was highest in beef, in both adults and children, primarily as a result of Pb levels. The ILCR was found dangerously high in milk for the children population due to high Pb levels. No significant differences in Pb ILCR levels were found in both adults and children for beef. Furthermore, significant differences (*P* < 0.05) were found in ILCR levels in Zn within the adult and children population consuming beef while in milk, significant differences exist in ILCR levels for Pb and Zn as shown in Table 3.

The target hazard quotient (THQ) in beef was found to be significantly low in Cu, Zn, and Fe, while this was highest in Pb (*P* < 0.05) in both adults and children. Very high Pb THQ led to elevated HI in beef. Also, THQ in milk was found to be significantly different in Cu and Zn (*P* < 0.05) in adults and children, and the HI in milk was found to be lower than that in beef as shown in Table 4.

## 4. Discussion

The study showed that levels of copper and zinc in milk and beef were comparably within the same range, and no cadmium was detected in all the samples. It had been thought that level of zinc was low in milk [17, 18], but this study provides evidence that milk in Uganda would be nutritious as beef. In addition, the additive advantage of copper in the milk would provide the local population with a cheap source of metalloids essential for physiological purposes [16, 19]. It is evident that these compounds accumulate in muscle and milk of the animals after consuming grasses in the area [38]. This is important since heavy metal concentrations in herbal distillates in Iran [39] show that heavy metal bioaccumulation occurs in animals following consumption of polluted fodder [40]. In Uganda, heavy metal contaminations in water [41] and fish [42] are indicators of environmental contamination, showing that the ecosystem in Uganda contains high levels of inorganic pollutants. Bearing in mind that micronutrients such as Zn, Cu, and Fe are important to the body [17–19], routine monitoring of these molecules in milk and beef would help to promote public protection [4, 5].

In Uganda, food safety assessment on milk and beef has been on microbial load [27, 28]; however, the current study shows that inorganic pollutants, which are more resistant to heat treatment, would pose a bigger public health problem to the general population. This is because long-term ingestion of livestock products, derived from livestock which graze on pastures polluted with heavy metals, would lead to development of major health hazards in man such as cancer, organ failure, and death [29].

It was also observed that significant differences in the levels of lead and iron existed in the beef and milk samples, with higher levels in the latter. High levels of iron are indicative of high hemoglobin content in the meat which seems to suggest that either the methods being used to slaughter the animals may be of low standard or it is an issue related to environmental contamination. We did not investigate further to determine the sources of these high levels of iron in the beef. However, muscles, due to their high metabolic activity, concentrate a lot of hemoglobin due to their increased demand for oxygen [20]. Beef continues to be an important source of iron, which would be of greater medical benefit to the local population in the management of anemia than milk.

It is also noteworthy that the concentrations of Pb in beef and milk were above US EPA recommended values. This implies that the ecosystem in which the animals graze is either naturally polluted with highly mineralized soil or there are man-made problems. Current scientific evidence seems to suggest that human activities are the major sources of environmental pollution with Pb, especially through the use of pesticides which are major sources of heavy metals [6, 8, 9, 43]. This is made worse by the inadequate management of industrial and agricultural wastes which contaminates the pastures. Heavy metal contamination of soil as a result of human activity is a real threat due to the ability of the plants to absorb these molecules, which then enter the food chain [6, 44]. This subsequently poses a major public health threat to both human and animal life.

Levels of Pb detected in beef were 10x above the recommended limit of 0.036 ppm [25]. In addition, the threat in children was higher due to their low body mass associated with poor xenobiotic metabolism in comparison to adults [31, 45]. The increased threat in the pediatric population which depends heavily on animal protein would inevitability put increased strain on the healthcare system since these require higher amounts of animal protein for growth and development [4, 5].

In Uganda, milk has been associated with large amounts of microbial load due to poor phytosanitary measures along the production chain in a majority of rural communities [27, 28]. Milk quality had not previously investigated for inorganic contaminants, and this study offers a baseline that would help improve the Dairy Development Authority (DDA) projects, with a goal of improving milk quality in Uganda [46].

The incremental lifetime cancer risk (ILCR) was high in beef as the levels of Pb played a major cumulative risk in increasing the cancer threat in the population. Furthermore, the hazard index (HI) was high (HI > 1), thus showing that the cumulative effects of contaminants in the samples would be dangerous. Bearing in mind that high Pb levels have been emphasized by this study in both milk and beef, future studies in Uganda would offer a more descriptive picture since this study was only conducted in Bushenyi district. The low trace elements are of physiological benefit to human, and reasons as to why they are in low concentrations in these samples would help guide policy, for improved consumer protection.

## 5. Conclusion

Observations made in this study show that chronic effects of the inorganic pollutants are a major public health threat, especially due to the strong cancer effects. Exposure to multiple contaminants results in additive and interactive effects; thus, the hazard index was used as an indicator of risk. Studies to include more areas in the region in order to determine the geographical extent of the threat at hand would have to be conducted; however, information in this study offers a probable cause to the increasing incidence of cancer within the Ugandan population, showing a need to revise current food safety policies and promote environmental protection.

## Figures and Tables

**Table 1:** Mean concentrations of heavy metals in milk and beef in Bushenyi district.

Heavy metal	Milk			Beef		
	*N*	Mean ± SEM	95% CI: (LL, UL)	N	Mean ± SEM	95% CI: (LL, UL)
Cu	15	0.56 ± 0.07^a^	0.44, 0.72	15	0.41 ± 0.06^a^	0.29, 0.53
Zn	20	43.72 ± 4.17^a^	34.92, 52.51	20	43.74 ± 5.20^a^	32.77, 54.71
Pb	18	10.48 ± 1.82^b^	6.62, 14.34	18	18.90 ± 2.40^a^	13.81, 24.00
Fe	20	11.96 ± 1.00^b^	9.85, 14.07	20	17.04 ± 1.15^a^	14.60, 19.49
Cd	20	nd	nd	20	nd	nd

Cu = copper, Zn = zinc, Pb = lead, Fe = iron, Cd = cadmium, nd = not detected, *N* = number of samples analyzed, CI = confidence interval, LL = lower limit, and UL = upper limit. Tukey's multiple comparison test with significant differences (*P* < 0.05) indicated by different superscripts (a, b).

**Table 2:** Estimated daily intake of pollutants in beef and milk of Bushenyi.

Parameters		Cu	Zn	Pb	Fe
		Mean concentrations × 10^−3^ (ppm/day)			
	TAL	0.5	1	0.0036	0.8
Adult beef EDI levels		0.001	0.096	0.053	0.037
Children beef EDI levels		0.001	0.134	0.074	0.051
*P* values of adults and children on beef levels		0.0986	0.0564	0.5013	0.0851
Adult milk EDI levels		0.001	0.0437	0.0109	0.0124
Children milk EDI levels		0.0018	0.1294	0.0322	0.0368
*P* values of adults and children on milk levels		0.001	0.001	0.002	0.001

TAL = tolerable allowable levels in humans; EDI = estimated daily intake. *P* values generated from *t*-test of the same inorganic compound.

**Table 3:** Cancer risk in Ugandan population.

Parameters	Pb	Zn	∑ ILCR
	Mean ILCR (×10^−4^)		
Adult beef ILCR levels	1.37^b^	0.0293^a^	1.399^b^
Children beef ILCR levels	1.92^b^	0.0410^a^	1.961^b^
*P* values of adults and children on beef levels	0.5013	0.001	—
Adult milk ILCR levels	0.283^a^	0.0133^a^	0.296^a^
Children milk ILCR levels	0.837^a^	0.0393^a^	0.876^a^
*P* values of adults and children on milk levels	0.002	0.001	—

ILCR = incremental lifetime cancer risk. US EPA comparisons indicated by superscripts (a, b). a = acceptable levels; b = unacceptable levels.

**Table 4:** Noncancer risk in Ugandan population.

Parameters	Cu	Zn	Pb	Fe	HI = ∑ THQ
	Mean target hazard quotient				
Adult beef THQ levels	0.00617	0.0977	4.09008	0.0158	4.210
Children beef THQ levels	0.00863	0.137	1022.5	0.02213	1022.7
*P* values of adults and children on beef levels	0.0986	0.0564	0.01292	0.0851	—
Adult milk THQ levels	0.00324	0.0513	2.116	0.00830	2.179
Children milk THQ levels	0.0096	0.1518	6.2648	0.0119	6.438
*P* values of adults and children on beef levels	0.001	0.001	0.0801	0.0690	—

## Abbreviations and Acronyms

AT: Period of exposure for noncarcinogenic effects
Cd: Cadmium
CDI: Chronic daily intake
CSF: Cancer slope factor
DDA: Dairy Development Authority, Uganda
EDI: Estimated daily heavy metal intake
EDtot: Exposure duration
FAO: Food and Agriculture Organization
Fe: Iron
ILCR: Incremental lifetime cancer risk
IR: Ingestion rate
Pb: Lead
RfD: Oral reference dose of the contaminant
THQ: Target hazard quotient using this equation
US EPA: United States Environmental Protection Agency
WHO: World Health Organization.
